# Critique of the Bias-of-Crowds Model Simply Restates the Model: Reply to Connor and Evers (2020)

**DOI:** 10.1177/1745691621997394

**Published:** 2021-09-15

**Authors:** B. Keith Payne, Heidi Vuletich, Kristjen B. Lundberg

**Affiliations:** 1 Department of Psychology and Neuroscience, University of North Carolina at Chapel Hill; 2 Department of Psychological and Brain Sciences, Indiana University Bloomington; 3 Department of Psychology, University of Richmond

Millions of people have taken an implicit test of racial bias, and the majority have displayed a preference for White people over Black people. What does it mean? The most common interpretation is that those who show such a preference are biased people and that they have an attitude, whether they explicitly acknowledge it or not, that favors White people over Black people. An alternative interpretation is that when people display a racial bias on an implicit test, it reflects the social environment they are in. It could be both. The lesson drawn from this research is important, because it bears not only on scientific theories of prejudice and discrimination but also on policy decisions about the best ways to eliminate racial disparities. Do we target individuals and try to change their attitudes? Or do we focus on social environments and the systems that impersonally preserve inequality?

In Payne et al. (2017), we argued that the field has focused too heavily on the individual attitude interpretation and that more can be learned by reconceptualizing implicit bias research to emphasize social environments. We argued that implicit bias reflects concept accessibility that varies as a function of both persons and contexts. But implicit bias measures are temporally unreliable at the person level and weakly associated with behavior. Aggregating across individuals reduces error variance and reveals more meaningful information in means, akin to the “wisdom of crowds” phenomenon (hence the “bias of crowds” name). Because of the statistical effects of aggregation, context-level effects have greater reliability and validity than person-level effects. The greater reliability and validity can be seen in the fact that regional levels of implicit race bias are associated with substantial disparities in health (Leitner et al., 2016; Orchard & Price, 2017), police use of force (Hehman et al., 2017), school discipline (Riddle & Sinclair, 2019), and other indicators of bias (Leitner et al., 2018; Rae et al., 2015).

Considering implicit bias as a context-level phenomenon not only improves measurement but also raises new and different research questions. Some examples include: What features in the environment cue biases? What are the historical forces that have led some places to be higher in bias than others? What structures and systems perpetuate biases over time? And how can environments be designed to reduce bias?

Connor and Evers (2020) took issue with our bias-of-crowds model, arguing for an “alternative view.” Curiously, their alternative view simply restated our model. The authors wrote, “Instead of reconceptualizing implicit bias as a feature of situations, this alternate view simply requires conceptualizing implicit bias as being an individual-level construct measured with substantial measurement error,” (pp. 1330–1331). They continued,

When enough noisy individual-level scores are aggregated, positive and negative measurement errors tend to cancel each other out, resulting in highly accurate measures of group means. Assuming that some real differences exist among group means (i.e., relatively more or less biased individuals clustering together in specific groups), this heightened measurement accuracy at the group level will tend to produce exactly the observations described by Payne and colleagues. . . . (p. 1331)

Their view thus appears to be identical to ours. We agree that implicit bias varies across persons and across situations. We agree that aggregating across individual scores reduces error variance to produce accurate situational means. And we agree that those accurate means produce stable estimates that correlate systematically with other variables.

So where is the disagreement? To the extent that there is any, it seems to be about what it means to be an “individual construct” or a “feature of situations.” In this reply, we first address the three empirical analyses reported by Connor and Evers. Then we consider the question of individuals and situations in the larger context of multilevel theorizing and measurement in psychology.

## Empirical Evidence

In one analysis, Connor and Evers generated simulated data representing implicit test scores for individuals nested in groups that varied in their mean levels of implicit bias. They showed that when the scores are aggregated, the test–retest stability and correlations with criterion measures were stronger for the aggregated groups than for the individual scores. This benefit of aggregation was stronger when the group sizes were larger (hence, more aggregation) and when the ICC was larger (hence, there were larger true context effects). This simulation provides evidence consistent with the view that implicit bias is a noisily measured construct at the individual level, which becomes less noisily measured and more strongly correlated with criterion variables when measured in the aggregate. Because this “alternative view” is identical to that posited by the bias-of-crowds model, we have nothing to dispute here.

In another analysis, Connor and Evers reanalyzed data from Vuletich and Payne (2019). In this study, which was itself a reanalysis of a study by Lai et al. (2016), nine experimental treatments were tested for their ability to modify implicit bias. The original Lai et al. (2016) study found that each of these manipulations was effective at reducing implicit bias on the immediate test, but none persisted to the follow up test. The result was interpreted as evidence that implicit attitudes are resistant to change, because participants ostensibly soon returned to their earlier level of bias. In the Vuletich and Payne (2019) reanalysis, we disaggregated results by college campus (the study took place across 18 campuses). Our approach found that campus contexts remained stable (*r* = .72) and that some campuses displayed consistently higher levels of bias than others. However, individual scores did not return rigidly to their earlier state, given that the test–retest correlation was only *r* = .25 at the individual level. Contrary to prior theorizing, individual levels of bias did not appear resistant to change. They changed a lot, but mostly randomly.

Vuletich and Payne (2019) reported a simulation in which subjects’ scores were randomly reshuffled into different campus groupings before aggregating the scores. When this was done, the large campus-level test-retest correlation was reduced to the size of the smaller person-level correlation. The point of this simulation was to demonstrate that mere aggregation does not spuriously create large correlations. Instead, there must be real differences between campuses to be revealed by aggregation. Connor and Evers reanalyzed these data and reported a “targeted expulsion” simulation. In this analysis, they removed participants with extremely high scores from high-bias campuses and extremely low scores from low-bias campuses, before aggregating them into campus means. Unsurprisingly, as progressively more extreme scores were removed, the test–retest correlation for campus means decreased. The authors take this result to mean that the stability of university-level means “completely relied on the stability of individual-level IAT scores” (p. 1342). But that conclusion does not follow.

The campus-level test–retest stability indicates that campuses with higher means at Time 1 tended to have higher means at Time 2. As we noted, there was some stable person variance (specifically, *r* = .25). Even a small amount of stable person variance is enough to reduce the campus-level stability in means if enough extreme scores are removed. Therefore, a process that removes the highest scorers from the high-bias campuses and the lowest scorers from the low-bias campus will obviously change the rank orders of the means. The authors claim that this reduction “completely relied” on the stability of individual scores. But individual scores also reflect stable context-level variance. People tend to inhabit the same contexts from one day to the next. For example, suppose that some students pass a confederate monument on their way to class each day, and this statue temporarily activates anti-Black implicit associations. Those who pass the monument might have 1 unit more bias when measured on any given day because of a consistent context effect. Picking them out as a high-bias score at Time 1 and deleting them from the Time 2 data set will remove part of the variance that made certain campuses consistently higher in bias.

Vuletich and Payne’s (2019) simulation demonstrated that there was nonzero variability in implicit bias across campuses. Connor and Evers’s simulation demonstrated that there was nonzero variation across people. Together, these simulations simply demonstrate that there are both person effects and context effects (as well as potential Person × Context interactions), which no one disputes.

Connor and Evers correctly noted that there was more variability between participants than between campus means. But individual-level variance displayed low reliability and validity correlations. From an individual perspective, it is mostly noise. The issue of variability at different levels of analysis highlights a statement in our original article that we now see was ambiguous and may be a cause of confusion. We wrote that “most of the systematic variance in implicit biases appears to operate at the level of situations” (p. 236). Our intended claim was not that there is *more variance* between situations than between individuals. Our intended claim was that the variance between situations is *more systematic* than the variance between individuals. By “more systematic,” we mean more reliable and valid, as evidenced by test–retest reliability and correlations with criterion variables.

In a third empirical demonstration, Connor and Evers reported an analysis of IAT scores from the Project Implicit website: There were small average differences in implicit bias scores across the days of the week. The highest levels of bias were observed on Sundays and the lowest on Fridays, and the rest were in the middle. Although these differences were very small at the level of individual scores, they were stable in the aggregate, producing a test–retest correlation of *r* = .95. And the aggregate weekday scores correlated strongly with aggregate weekday explicit bias scores on a feeling thermometer measure, *r* = .86.

Connor and Evers did not argue that this result contradicts any premises of the bias-of-crowds model. Instead, having dubbed it “the bias of weekdays,” they apparently thought that it was such a preposterous finding that it functions as a reductio ad absurdum. They wrote,

So if what matters for how we conceptualize implicit bias is the level at which we can observe the greatest reliability and criterion correlations, then we must conclude that most of the systematic variance in implicit bias is at the weekday level. We hope that readers agree that this would be a strange conclusion. (p. 1332)

We do not agree. The weekday effect might be meaningless, because it is post hoc and not motivated by any coherent theory. And yet, if it turned out that across the hundreds of millions of people in the U.S., racial discrimination was slightly more common on Sundays than Fridays, and this could be predicted by daily implicit bias scores, then it could be very important indeed.

The authors attached a great deal of importance to the small effect of weekdays on individual scores, emphasizing that individuals’ previous scores account for “600 times more” variance than weekday does. The difference is small in absolute terms (6% versus .01%). But more important, this comparison is not relevant to evaluating bias-of-crowds model. The effects of context on individual scores might be large or small (and in this case, the authors invented this effect as an example of a very small effect). Context-level effects should be used to make inferences about contexts. As long as the conclusions are drawn at the same level of analysis as the data are aggregated—the county, campus, or even weekday—aggregated scores can reveal potentially important information about the contexts. This difference of opinion in what counts as meaningful leads us to consider the implications of this debate for multilevel measurement in psychology more generally.

## Signal, Noise, and Multilevel Measurement

Connor and Evers’s argument that implicit bias is really an individual construct seems to rest on an essentialist assumption that applies to individuals but not to situations: if scores vary systematically across people, they reflect an “individual construct.” But if scores vary systematically across situations, they do not reflect “features of situations.” Connor and Evers argued that researchers should reduce measurement error to better measure individual attitudes. But they do not think reducing measurement error by aggregating across individuals allows researchers to measure situations. Perhaps we disagree about what systematic and error variance mean. Here is our view.

In a deterministic universe, error variance is not random in the sense that it is uncaused; instead, it is random in the sense that we cannot account for all of the causes operating. Any test score reflects the construct of interest plus many other factors that are not known or not modeled. In some cases, what counts as “error variance” depends on the nature of the construct. For a stable trait-like construct, unstable test scores may reflect poor measurement. For a changeable state-like construct, however, test scores that are too stable may reflect poor measurement (Cronbach, 1951).

In other cases, what counts as error variance depends on the goals of the researcher. To test individual difference hypotheses, researchers average across situational influences because they consider the individual factor to be the signal and the situational factors to be the noise. Likewise, when researchers run an experiment, they average across individual differences to focus on group means, considering individual differences to be noise. Any given measure reflects influences of persons, situations, their interactions, and other unknown factors in countless combinations. So researchers simplify by aggregating across factors that are of less interest for their purposes. Aggregation is always a part of psychological measurement. And how we aggregate influences what we measure. Consider three examples where aggregated measurements reveal something new and different from individual measures.

Emotion can vary at both the person level and the context level. Some people, for example, are more prone to fear than others. But if the average level of fear for people coming out of a certain movie is systematically higher than in other contexts, it is reasonable to conclude that the movie is scary. It makes little sense to insist that because fear is really an individual construct, movies, rollercoasters, or haunted houses cannot be scary.

To take another example commonly used to teach multilevel models, the academic scores of students in different schools can be used to measure not only the performance of the students but also the performance of the schools. When student scores are aggregated to measure school performance, researchers can ask meaningful questions about the schools. For example, they might examine the relationship between school-level performance and the poverty rates, property-tax rates, or racial segregation in the school districts. These factors affect school performance beyond the traits of individual students.

A final example: Radioactive decay happens when the nucleus of an unstable atom disintegrates into a more stable element, releasing energy in the process. At the level of individual atoms, the process is random. No one can tell which atom is going to decay and when. But in the aggregate, decay rates are extremely stable, so that radioactive elements have well-known half-lives. This stability is useful. Even small bits of radiocarbon, for example, can be used to reliably measure the age of rocks, fossils, or prehistoric skeletons. One might argue that radioactive decay is really an atom-level process, so an element’s half-life is not interesting because it is simply the statistical result of aggregation. But that would miss opportunities to answer interesting questions. It is good science to ask questions at the same level of analysis that one can find reliable and valid answers.

In the same way, aggregate effects can tell us about the level of racial bias in contexts with great accuracy. They can tell us, for example, that Black residents are more likely to be shot by police in some cities than others. Because this relationship is fairly strong (Hehman et al., 2017), a simple linear equation lets us forecast racial disparities in a new city on the basis of average implicit bias scores with good accuracy. As with individual atoms, we would not know who is going to pull the trigger or on which day. But for many purposes, such as understanding patterns of systemic racism and considering citywide or countrywide policy solutions, the context is important.

How do we know if the context-level measurements are meaningful? Through empirically validating the measures, just as we do for person-level measures. In the past few years, researchers have made progress toward establishing the validity of context-based measurements. Hehman et al. (2019) reported evidence for the validity of implicit bias measured at the city, county, and state levels. They found that aggregate measures were correlated with variables theoretically expected to be related and were not correlated with irrelevant variables.

In our own research, we found that county-level implicit biases were substantially correlated with the proportion of the population enslaved in the 1860 census (Payne et al., 2019). Counties more dependent on slavery in 1860 have greater residential segregation, greater racial disparities in poverty, and greater disparities in upward mobility today. These contemporary markers of systemic racism statistically mediated the association between slavery and implicit bias today. In Vuletich and Payne (2019), we found that an index of structural racism was strongly associated with campus-level implicit bias. We believe these findings help validate contextual variation in implicit bias as meaningful and important. Some places really are more biased than others (Murphy et al., 2018; Murphy & Walton, 2013).

Nearly any variable can be analyzed at the person level or at the context level (or both). The comparative advantages depend on signal and noise at each level. Aggregation reduces error variance that is randomly distributed, but it does not reduce systematic error. Aggregation can improve measurement only if the measure has some validity to begin with. In the case of implicit bias, the relative advantages of the context-level analysis are clear because measures are so unreliable at the person level. Researchers who care only about person-level effects can typically expect unstable data and validity correlations in the range of .20, explaining less than 5% of variance in behavior. Context effects—whether regional or experimental—afford different questions and usually answer them more accurately.

We framed the bias-of-crowds model around three findings that are puzzling from a traditional individual difference view. First, individual implicit bias scores are unstable, but aggregate scores are very stable. Second, despite low stability, children show implicit bias effects indistinguishable from those of adults. And third, scores are weak predictors of criterion variables at the person level but strong predictors at the context level. We argued that these puzzles are naturally solved in the bias-of-crowds perspective, because aggregation reduces the random variance of individual scores to reveal meaningful context effects. Children and adults share the same contexts, which themselves are stable and associated with important outcomes. Connor and Evers argued that these puzzles are not really puzzling because they can be explained by the statistical effects of aggregation across contexts. Once again, this simply repeats the argument we made in the 2017 article. Puzzles rarely seem puzzling once the answer has been explained (Roese & Vohs, 2012).

Much research remains to be done. But after 25 years of research on implicit bias as an individual attitude, these context-based questions are only now beginning to be asked. The power of applying a new model to a well-established phenomenon is that a conceptual shift can spark new questions and new insights.

Ultimately, there is a rigidity in Connor and Evers’s argument that just because scores are affected by situations, that does not make them a measure of situations. To the contrary, any test becomes a measure of situations if it is affected systematically by situations. One of the endlessly inspiring things about science is that observations can be used in new ways to answer questions that had not been asked before. That is why the pattern of wear on a museum floor can be used to measure the popularity of different paintings (Webb et al., 1981). It is why betting markets can be used to predict the future (Arrow et al., 2008). And it is why light can be used to measure not only the brightness of stars, but even the expansion of the universe (Riess et al., 2005). One can insist that these observations are *really* measures of nothing more than foot friction, individual ignorance, and luminance. But to do so is to miss out on some wonderful insights.

## Transparency

*Action Editor:* Laura A. King

*Editor:* Laura A. King
